# Genetic monitoring on the world’s first MSC eco-labeled common octopus (*O. vulgaris*) fishery in western Asturias, Spain

**DOI:** 10.1038/s41598-023-29463-6

**Published:** 2023-02-15

**Authors:** N. Pirhadi, M. Parrondo, A. Romero-Bascones, R. Thoppil, J. L. Martínez, M. P. Fernández-Rueda, I. Márquez, L. García-Flórez, E. Dopico, T. Pérez, Y. J. Borrell

**Affiliations:** 1 Department of Functional Biology, Genetics, University of Oviedo, 33006 Oviedo/Uviéu, Spain; 2 Sequencing Unit, Biotechnical and Biomedical Testing, Scientific‐Technical Services, University of Oviedo, 33006 Oviedo/Uviéu, Spain; 3 Directorate General of Maritime Fisheries (DGPM), CEP Fisheries Experimentation Centre, Regional Ministry of Rural Development and Natural Resources from the Principality of Asturias, 33212 Gijón/Xixón, Spain; 4 Animal Health Area, SERIDA, 33394 Gijón/Xixón, Spain; 5 Department of Education Sciences, University of Oviedo, 33005 Oviedo/Uviéu, Spain

**Keywords:** Population genetics, Marine biology, Ecological genetics

## Abstract

*Octopus vulgaris* (Cuvier, 1797) is a cephalopod species with great economic value. In western Asturias (northwest of Spain), *O. vulgaris* artisanal fisheries are relatively well monitored and conditionally eco-labeled by the Marine Stewardship Council (MSC). Despite this, the Asturian octopus stocks have not been genetically assessed so far. In order to improve the current fishery plan and contrast the octopus eco-label validity in Asturias, 539 individuals from five regions of the *O. vulgaris* geographic distribution, including temporal samplings in Asturias, were collected and genotyped at thirteen microsatellite loci. All the samples under analysis were in agreement with Hardy–Weinberg expectations. Spatial levels of genetic differentiation were estimated using *F*-statistics, multidimensional scaling, and Bayesian analyses. Results suggested that the *O. vulgaris* consists of at least four genetically different stocks coming from two ancestral lineages. In addition, temporal analyses showed stability in terms of genetic variation and high N_E_ (> 50) for several generations in different localities within Asturias, pointing out to indeed sustainable fishery exploitation levels. Even though, the current Asturias fishery plan shows no significant genetic damages to the stocks, the regional-specific management plans need systematic genetic monitoring schemes as part of an efficient and preventive regional fishery regulation strategy.

## Introduction

Marine biological resources are limited, yet the global increasing demand for seafood products has led to the overexploitation of fisheries, which is highlighted as one of the current main threats to marine species. It has been argued that the fraction of fish stocks that are within biologically sustainable levels decreased by more than a 30% from 1974 to 2017, whereas the percentage of stocks fished at biologically unsustainable levels increased, especially in the late 1970s and 1980s, from 10% in 1974 to 34.2% in 2017^1^. Although overfishing is undoubtedly the greatest threat to marine biodiversity^2–4^, it is clear that the depletion of the world’s fish stocks cannot be attributed solely to fishing. Habitat destruction^5,6^, pollution^7,8^, anthropogenic climate change^9^ or invasive species^10^ also have an impact on fish populations. However, the high economic growth observed in recent years has triggered a global increase in consumption, which in turn has had a damaging effect on the natural environment^11^ and the subsequent loss of the ocean biodiversity^12^. Fortunately, over the past few decades there has been an increasing trend in global awareness regarding this emerging issue^12^, prompting several proposed programs for establishing sustainable fishery plans^13–15^ as well as the development of tools that can educate consumers about the impact of products on the natural environment throughout their life cycle, but which at the same time can also provide producers with the opportunity to inform consumers about the benefits of their products^11^.

Eco-labels are “seals of approval” given to products that are deemed to have low or no negative impacts on the environment^16^. The FAO recognized that eco-labels could contribute to improved fisheries management and convened a technical consultation in 1998, which led to the development of the “Guidelines for the Ecolabelling of Fish and Fishery Products from Marine Capture Fisheries”^17^. Since then, numerous programes have been proposed for eco-labeling seafood products in an effort to encourage fisheries managers to create sustainable fisheries^16^. These initiatives aim to provide a market-based incentive for sustainable fisheries management. Processors, wholesalers, and retailers who purchase products from these accredited fisheries can acquire the right to affix an eco-label, informing consumers that the product has been caught in a sustainable fishery. Hypothetically, if there were a demand for environmental quality, consumers would respond by purchasing those products with an eco-label, thereby reducing demand for those without and causing price devaluation on unlabeled products. This may result in fishermen putting pressure on fisheries managers to achieve sustainability accreditation and thus receive a higher percentage of the price^16^. One of the most recognized and prestigious ecolabels in use today is the Marine Stewardship Council (MSC).

Designing sustainable fishery management requires deep understanding of the current conservation status of species and valid biological data. Stock assessment is crucial, but it can be challenging due to methodology difficulties, financial cost and intensive data requirements, so its application is usually limited to industrial fisheries^18^. Effective and sustainable management plans for artisanal fisheries also need to be established to reduce potential threats to marine resources and ensure environmental protection and sustainability^19,20^. Unfortunately, the vast majority of fished artisanal stocks are unassessed^21,22^, and their status, although highly uncertain, is generally considered worse than that of data-rich populations^23,24^.

Stock has been historically defined as an intraspecific group of randomly mating individuals with temporal and spatial integrity^25,26^. Defining stock boundaries for fisheries is fundamental and it is linked to the central idea that each stock has a harvestable surplus, and fisheries that comply with this limit will not compromise the stock’s natural perpetuation. Stocks that are scientifically assessed are usually in better condition than stocks that are unassessed^18,27^, being either exploited at sustainable rates or being re-built. Since January 2014, the reformed Common Fisheries Policy (CFP) of the European Union prescribes the end of overfishing and the rebuilding of all stocks above levels that can produce maximum sustainable yields (MSY). Although the global number of sustainable fishery products is increasing, there is reported evidence that the types of data upon which most assessments are established can be insufficient and misleading^22,28,29^. In this matter, there is an emerging need for radical changes in monitoring and collecting precise biological data from marine stocks; moreover, there is a direct link between fishing pressure and loss of species gene pools^30,31^. Genetic factors play an important role in fishery resource conservation because the latter are the product of their genes, the environment, and the interactions between the two^32^. Failure to detect biological characteristics of a stock within a population can lead to genomics changes and the subsequent loss of populations genetic diversity^33^.

Traditionally, fisheries conservation and management have been based on abundance data, productivity estimates, and information on stock dynamics^34,35^. However, genetics offers a diverse collection of versatile and useful tools to inform fisheries management on issues that have a biological basis^36^. Despite this, genetics has not yet been effectively integrated into fishery management schemes mainly due to the existing gap between managers, decision-makers, fisheries scientists and geneticists, resulting in less application of fisheries genetics^37^. Significant progress and findings in the field of marine genomics as well as the proven importance and validity of molecular genetic data, have made them vital tools for species identification; fisheries stock structure; resolving mixed-stock fisheries; age biomarkers; ecosystem monitoring; estimating harvest rates and abundance; genetic diversity, population abundance, and resilience; evolutionary responses to fishing; genetic effect of stock enhancement; detection of pathogens and invasive species; and product provenance and fisheries surveillance^36^.

The use of molecular genetic techniques in fisheries research has increased dramatically in the last few decades. Microsatellites and mitochondrial DNA markers have been used at an increasing rate in fisheries and today the application of new techniques to fisheries research, such as mass sequencing-derived markers, has grown significantly^38^. However, there is a clear species bias where marine invertebrates continue to lack genetic and genomic resources compared to other widely studied groups such as fish^39^. The importance of accurate genetic data is even more tangible when it comes to marine resources with huge market interest such as *Octopus vulgaris* Cuvier, 1797.

*O. vulgaris*, also known as common octopus, is a cephalopod species with a very strong market interest in Europe, and specifically in Spain, the current major supplier of octopus in the global seafood market^40,41^. The great commercial interest in *O. vulgaris* along with the growing demand for this species, increase the potential risk of fundamental changes in its population structure, which can lead to stock collapse due to overexploitation. Recent studies have reported a significant decrease in octopus global captures, mostly caused by unmonitored fishing activities in some regions^42^. Particularly, the information obtained from the analysis of genetic markers in this species can be useful in assessing the level of the marine stock healthiness, and subsequently in framing more sustainable fishery plans^43^. It is vitally important that this valuable species is sustainably managed, as it is not only an important food but also provides a significant source of income to local communities and families^31^.

A co-management system was established for the small-scale fishery (SSF) of octopuses with baited traps in Asturias (NW Spain) from 2000 to 2001 covering from the Eo estuary to the Nalon estuary^44^. The management measures include limiting entry to licensed boats from any of the eight legally recognized fishers’ associations, seasonal closures (almost always between mid-July to mid-December), type and number of gears and boat regulations, minimum landing octopus weight of 1 kg, and a maximum catch per season^44,45^. In the last two fishery seasons, Asturias management set the annual global catch as equal to the mean latent productivity (instead of the Maximum Sustainable Yield (MSY) criteria) minus two times the standard error of the estimate as a precautionary, sustainable and economically viable annual harvest rate^46^.

The artisanal octopus fishing with traps in western Asturias was certified with the Marine Stewardship Council (MSC) eco-certification in 2016, which made it the world’s first cephalopod fishery with this accreditation. However, the BUREAU VERITAS IBERIA (the certifying body) noted some weaknesses when recommending the MSC sustainability certificate for this fishery. They reported that “biological information on the resource was still scarce”, explicitly recommending that “information on the knowledge of octopus populations need to be improved”^47^. Having passed the first 5 years of its initial certification, and with explicit use of a precautionary annual harvest rate^46^, the octopus fishery of western Asturias has achieved MSC recertification in 2021. In the case of the western Asturias octopus fishery, the eco-label validated the fishery as sustainable and environmentally friendly since it has a minimum impact on the marine ecosystem^48^.

Over the past twenty years, some research has been conducted on studying the Atlantic *O. vulgaris* population using DNA markers^31,49–51^. However, the genetic status of *O. vulgaris* stocks in the southern area of the Bay of Biscay is still poorly studied. In this work, the main objectives have been to determine, both spatially and temporally, the levels and characteristics of genetic variation in octopus samples from areas localized within the MSC-certified octopus management plan in western Asturias, in comparison with other areas of the species geographic distribution, as well as to assess the stability of gene frequencies and carry out the first estimations of effective population sizes for this valuable species. All this data can address questions of direct relevance for the sustainable management of the species.

## Results

**Figure 1:**
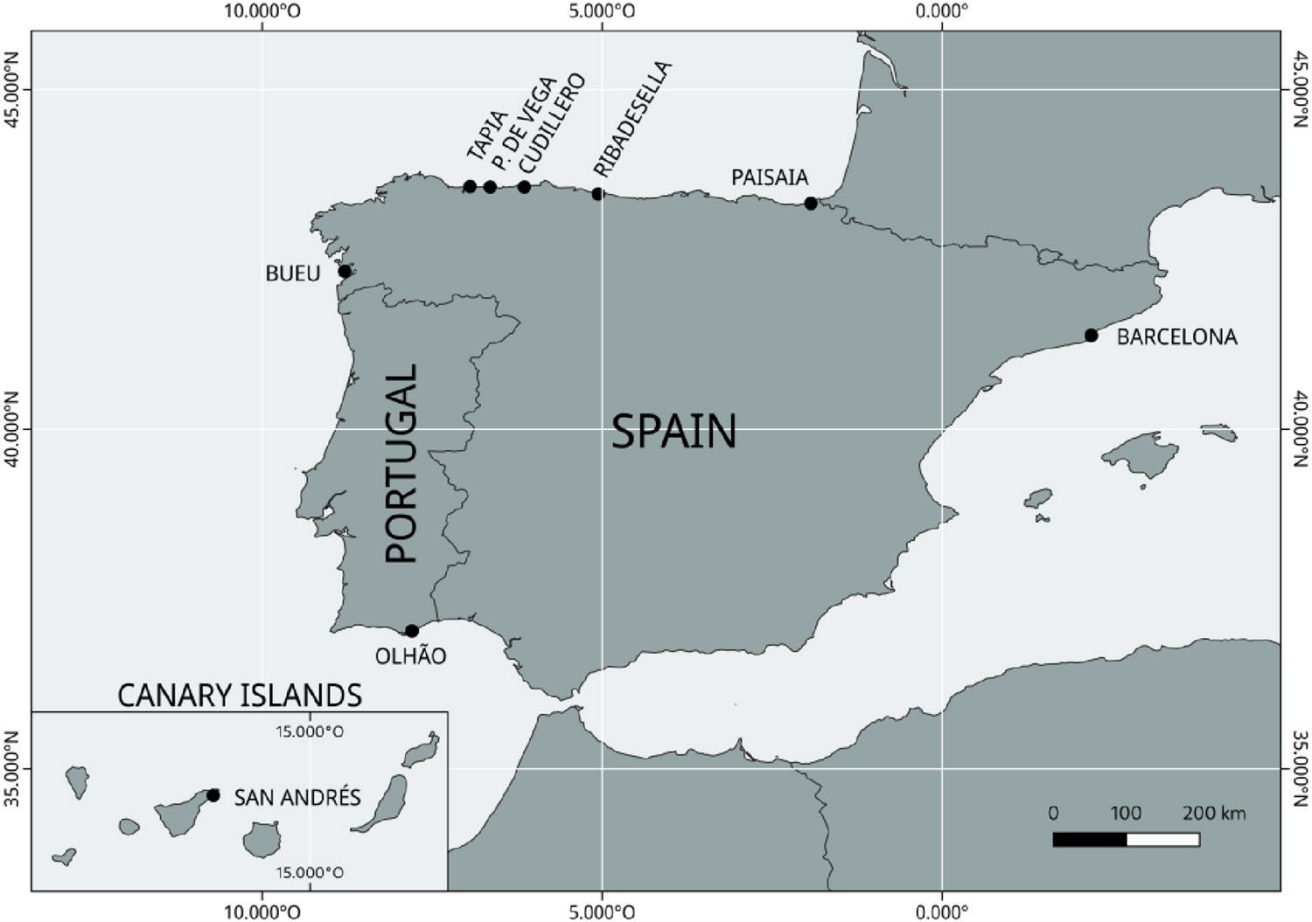
Study regions of the Iberian Peninsula and Canary Islands for genetic analyses of *O. vulgaris* using microsatellites. A total of 9 localities were sampled (black dots) for population genetic analyses.

In this work, we have conducted temporal and spatial genetic analysis in *O. vulgaris* samples from several areas of the species’ geographic distribution (Basque Country, Asturias, Galicia, Algarve, Canary Islands, and Catalonia) and including localities where the MSC-certified western Asturias octopus fishery is established (i.e. Tapia de Casariego, Puerto de Vega and Cudillero; Fig. 1).

### Microsatellites genetic variation in common octopus (*O. vulgaris*)

A total of 13 microsatellite loci were arranged into 2 multiplex PCRs (M1, M2) (Table 1). The number of alleles per locus (k) varied from 3 to 31 between loci, with an average of 13.77, and yielded an average allelic richness (A_R_) of 7.03 (ranging from 1.36 to 15.65) (Table 1). The observed heterozygosity and the within-population gene diversity across loci ranged from H_O_ = 0.04 (M1; Vulg12) and H_S_ = 0.06 for the same locus, to H_O_ = 0.89 (M2; OV10) and H_S_ = 0.92 (M1; OCT08). The global averages for observed heterozygosity and within-population gene diversity were 0.49 and 0.51, respectively (Table 1). Only the marker OCT08 (*F*_IS_ = 0.113 p < 0.05), showed significant deviations from the Hardy–Weinberg equilibrium (13 loci average *F*_IS_ = 0.04 p < 0.05) whereas the other twelve loci (92%) agreed with Hardy Weinberg expectations (Table 1). The potential presence of null alleles (Brookfield 1 statistic q > 0.05) was detected for only four loci (31%), specifically for the locus OCT08 (Brookfield 1996, B = 0.059); Vulg15 (B = 0.114); Vulg12 (B = 0.056) and Ovul08 (B = 0.090) (Table 1). The mean overall *F*_ST_ values for the 13 microsatellites was *F*_ST_ = 0.07 (p < 0.05) and four of them showed higher and significant *F*_ST_ values: Vulg15 (*F*_ST_ = 0.340, p < 0.05); Vulg12 (*F*_ST_ = 0.469, p < 0.05); Vulg13 (*F*_ST_ = 0.083, p < 0.05); Ovul08 (*F*_ST_ = 0.260, p < 0.05) (Table 1). The analysis conducted with BayeScan v2.1 for outlier detection resulted in no loci under selection or biased by species admixture and hybridization, which have the same expectations in terms of outliers; however, when we found 1 locus with positive α-values, the q-value was higher than 0.05 and not fall apart from neutral expectations for the analyzed genetic data.

**Table 1:**
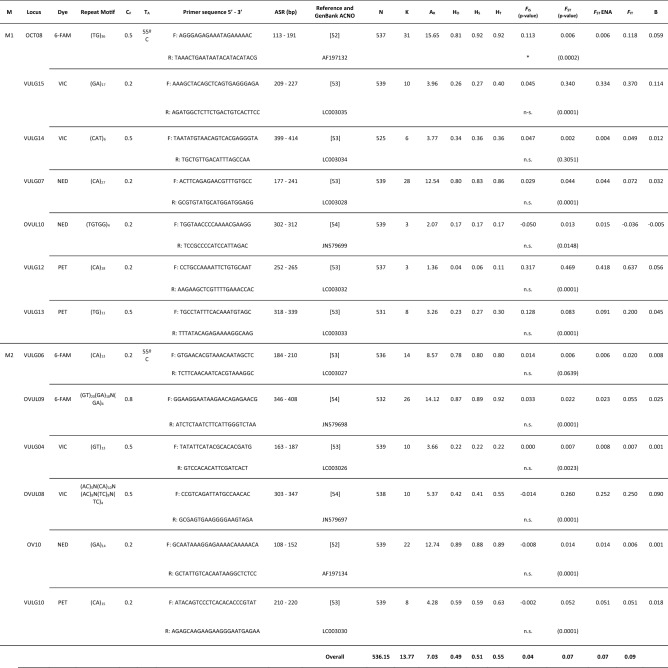
Overall microsatellite information based on multiplex PCRs typifying *O. vulgaris* populations coming from 9 different localities of the Iberian Peninsula and the Canary Islands.

*M* multiplex, *C*_*F*_ PCR final concentration, *T*_*A*_: annealing temperature, *ASR* Allele size range in base pairs, *REF* reference where markers are reported, *N* sample size, *k* number of alleles per locus, *A*_*R*_ allelic richness for the minor possible number of diploid individuals by sample (n = 23), *H*_*O*_ observed heterozygosity, *H*_*S*_ within population gene diversity, *H*_*T*_ overall gene diversity. Weir and Cockerham^55^ *F* statistics: *F*_IS_, *F*_ST_, *F*_ST_ ENA (excluding null alleles following Chapuis and Estoup^56^) and *F*_IT_. B: Brookfield 1 statistic for null allele’s inferences.

The comparative analyses for levels of genetic variation among samples by locations revealed Olhão (Algarve) showing the highest values for allelic richness (7.621) while Cudillero (Asturias) showed the highest number of private alleles for the 2020–2021 fishery season (Ap = 7) (Table 2). Significant differences in allelic richness and observed heterozygosity were found in this work (p < 0.05) ranging from A_R_ = 6.079 (Pasaia, Basque Country, Spain; 2020–2021) to A_R_ = 7.621 (Olhão, Algarve, Portugal; 2020–2021) and from H_O_ = 0.444 (Puerto de Vega, Asturias, Spain; 2020–2021) to H_O_ = 0.571 (Olhão, Algarve, Portugal; 2006–2007) (Table 2). The expected heterozygosity was also significantly differentiated (p < 0.01) among the samples and San Andrés (Canary Islands, Spain; 2020–2021) showed the highest values (H_E_ = 0.613) whereas Cudillero (Asturias, Spain; 2020–2021) showed the lowest ones (H_E_ = 0.461) (Table 2). Comparative temporal analyses revealed no significant differences in the levels of genetic variation during the past 14 years (2007–2021) (approximately 9 generations, considering the life-span of 1.5 years for *O. vulgaris*). That was the case of Puerto de Vega (Asturias, Spain) and Olhão (Algarve, Portugal) and the same non-significant result was also obtained for the time range between 2018 and 2021 for the Ribadesella, Cudillero, Puerto de Vega, and Tapia de Casariego populations (Asturias); reflecting stable levels of genetic variation for the stocks in these locations for the mentioned period (Table 2).

**Table 2:** Levels of genetic variation after spatial and temporal genetic analyses using microsatellites in *O. vulgaris* from the Iberian Peninsula and the Canary Islands.

FAO Fishing area	Region	Locality	Fishery season	Code	N	N_A_	A_P_	A_R_	H_O_	H_E_	*F*_IS_ (HWE)	TPM p
Bay of Biscay—Central (Division 27.8.b)	Basque Country (Spain)	Pasaia (PS)	2020–2021	21PS	24	6.615	0	6.079	0.501	0.513	0.024 (n.s)	0.420
Bay of Biscay—South (Division 27.8.c)	Asturias (Spain)	Ribadesella (RB)	2017–2018	18RB	38	8.000	10	6.454	0.471	0.501	0.060 (n.s)	0.878
			2020–2021	21RB	27	7.230	4	6.353	0.476	0.495	0.040 (n.s)	0.953
		Cudillero (CU)	2017–2018	18CU	37	7.923	5	6.401	0.476	0.484	0.018 (n.s)	0.936
			2020–2021	21CU	40	7.923	7	6.195	0.465	0.461	− 0.007 (n.s)	0.984
		Puerto de Vega (PV)	2006–2007	07PV	46	8.307	21	6.342	0.452	0.472	0.042 (n.s)	0.953
			2017–2018	18PV	39	8.076	8	6.670	0.489	0.490	0.003 (n.s)	0.878
			2020–2021	21PV	32	7.230	3	6.153	0.444	0.464	0.046 (n.s)	0.420
		Tapia de Casariego (TP)	2017–2018	18TP	37	7.846	7	6.494	0.465	0.483	0.036 (n.s)	0.658
			2020–2021	21TP	31	7.384	1	6.267	0.467	0.465	− 0.003 (n.s)	0.632
Portuguese Waters—East (Division 27.9.a)	Galicia (Spain)	Bueu (BU)	2020–2021	21BU	38	8.307	1	6.873	0.468	0.489	0.043 (n.s)	0.892
	Algarve (Portugal)	Olhão (OL)	2006–2007	07OL	44	8.692	26	7.179	0.571	0.591	0.034 (n.s)	0.892
			2020–2021	21OL	40	8.923	5	7.621	0.570	0.588	0.031 (n.s)	0.916
Canaries/Madeira Insular (Division 34.1.2)	Canary Islands (Spain)	San Andrés (SA)	2020–2021	21SA	28	7.461	2	6.496	0.541	0.613	0.121 (n.s)	0.500
Balearic (Division 37.1.1)	Catalonia (Spain)	Barcelona (BC)	2020–2021	21BC	38	8.769	6	7.318	0.551	0.565	0.025 (n.s)	0.960

*N* sample sizes, *N*_*A*_ Mean number of alleles by locus, *A*_*P*_ Private alleles (calculated spatially for the same fishery season), *A*_*R*_ Allelic richness for the minimum possible number of diploid individuals per sample, *H*_*O*_ observed heterozygosity, *H*_*E*_ expected heterozygosity, *F*_*IS*_ degree of departure from expected Hardy–Weinberg proportions within samples (*n.s* not significant), *TPM p* Wilcoxon sign test probability under TPM method.

*p < 0.05, **p < 0.01, ***p < 0.001.

The highest level of discrepancies between observed and expected heterozygosity was noticed in San Andrés (Canary Island, Spain) but none of the samples disagreed with Hardy Weinberg expectations (Table 2). The population bottleneck hypothesis was tested with the software Bottleneck and no significant excess of predicted heterozygotes was observed under the TPM model. Results from Wilcoxon’s test showed no sign of significant recent bottlenecks in the nine populations under study (Table 2).

### Spatial and temporal genetic analyses in common octopus (*O. vulgaris*)

**Figure 2:**
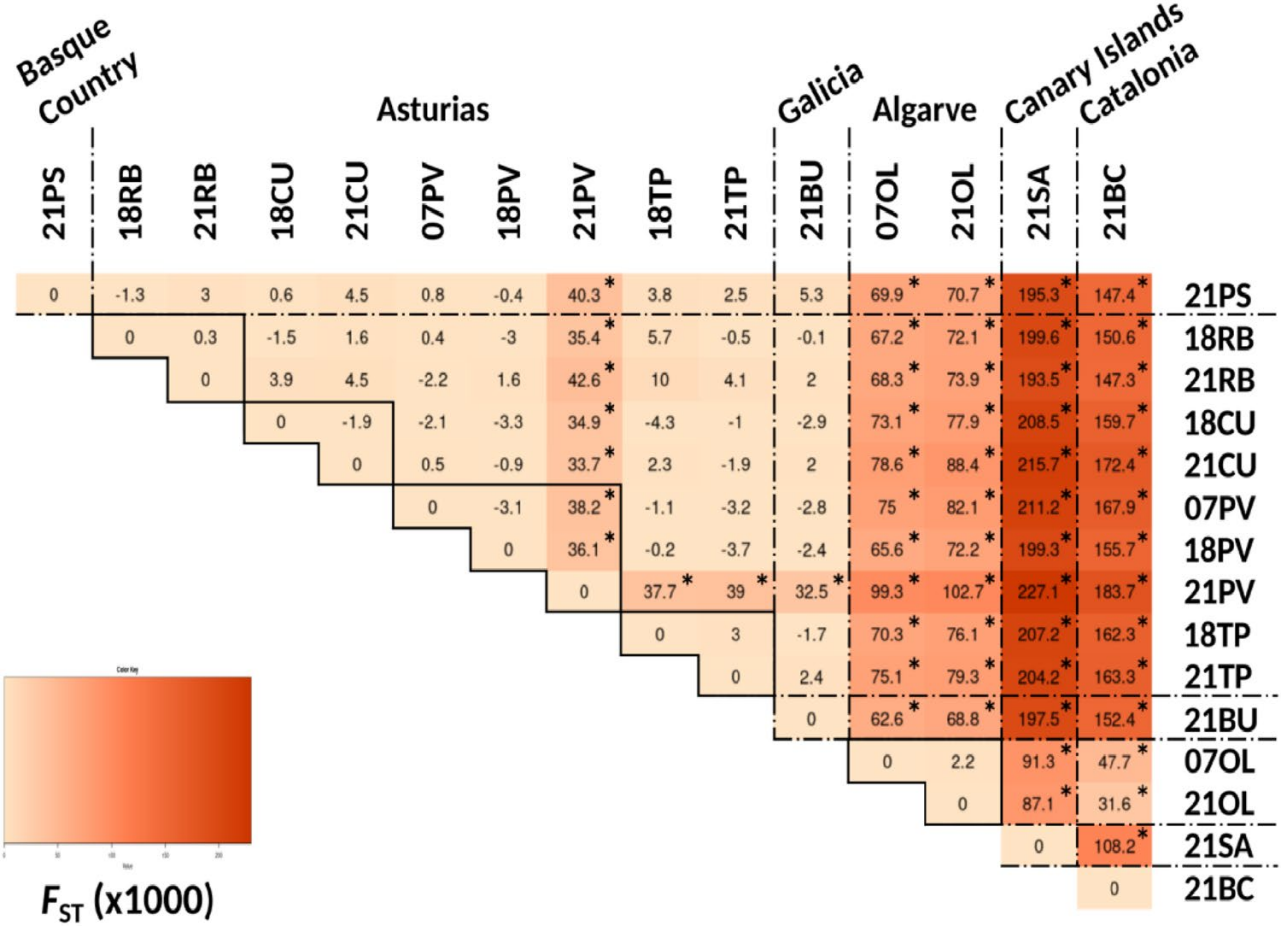
*F*_ST_ heatmaps (based on Weir and Cockerham^55^) following genetic analyses of *O. vulgaris* using microsatellites along the Iberian Peninsula and the Canary Islands. Labels indicate the fishery season (first two digits) and the locality (Pasaia (PS), Ribadesella (RB), Cudillero (CU), Puerto de Vega (PV), Tapia de Casariego (TP), Bueu (BU), Olhão (OL), San Andrés (SA), Barcelona (BC)). The darker the color, the higher the *F*_ST_ value. Asterisks indicate significant p-values (p < 0.05) after Bonferroni correction. Temporal comparisons are highlighted by black rectangles.

**Figure 3:**
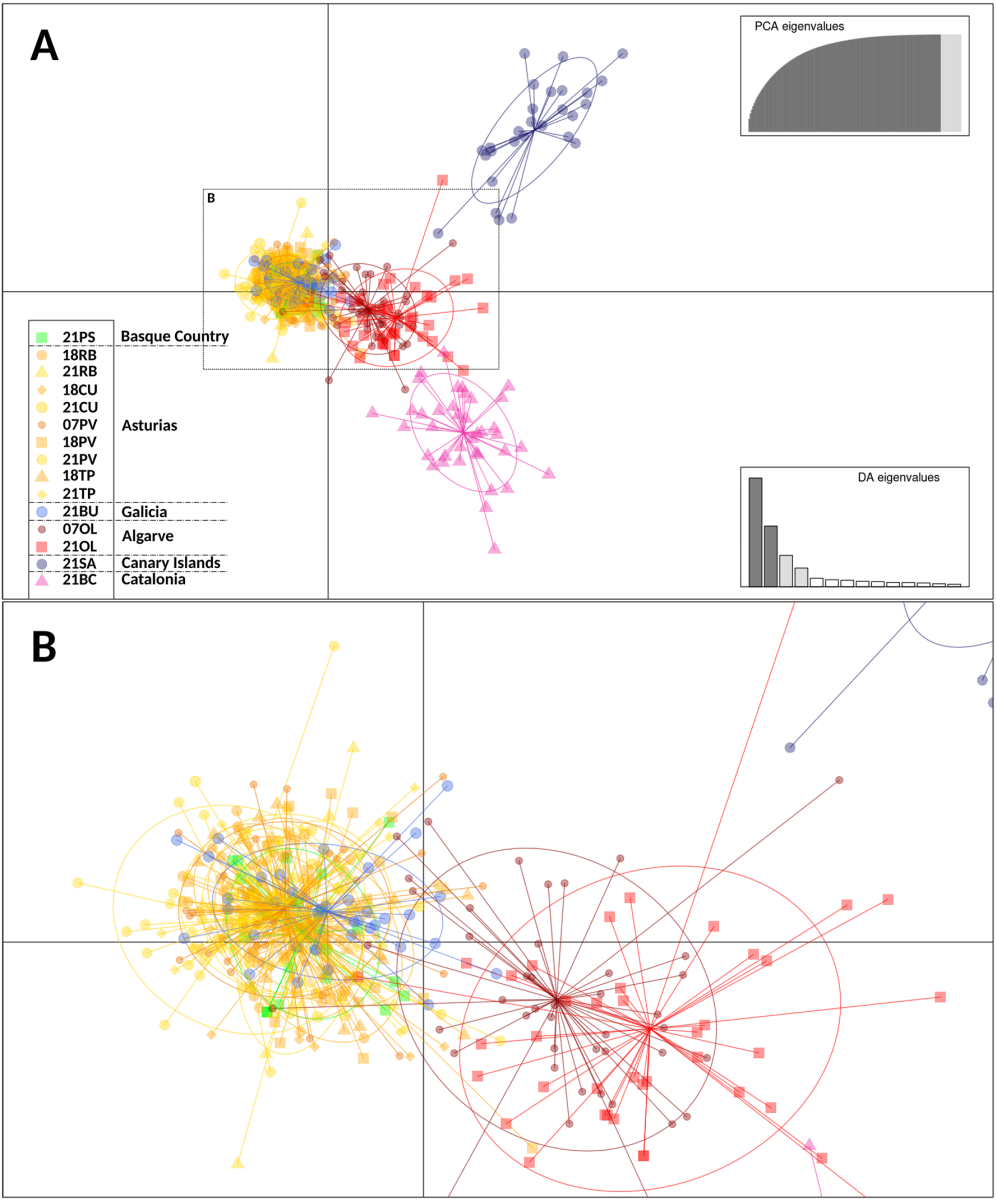
Genetic clustering using Discriminant Analysis of Principal Components (DAPC) of *O. vulgaris* populations using microsatellites along the Iberian Peninsula and the Canary Islands. (**A**) Global analysis, (**B**) Enlarged detail of localities in the Basque Country, Asturias, Galicia and Portugal. Labels indicate the fishery season (first two digits) and the locality (Pasaia (PS), Ribadesella (RB), Cudillero (CU), Puerto de Vega (PV), Tapia de Casariego (TP), Bueu (BU), Olhão (OL), San Andrés (SA), Barcelona (BC)).

A significant spatial structuring pattern was found in this work for *O. vulgaris* samples (Fig. 2). The pairwise *F*_ST_ values revealed high genetic differentiation among samples from four separated geographic areas: (1) Bay of Biscay (including Basque Country and Asturias) and also the northern Atlantic samples from Galicia; (2) Southern Portugal; (3) Canary Islands and 4) Catalonia (Figs. 2, 3). Within the Bay of Biscay and adding the sample from Bueu (Galicia, 2020–2021) we found genetic homogeneity by *F*_ST_ values, except for one sample from Puerto de Vega (Asturias, Spain; 2020–2021), showing a significant differentiated genetic pattern with the rest of the samples (Fig. 2). These patterns of genetic differentiation increased with geographic distance between locations. The results of the partial Mantel tests indicated a correlation between genetic and geographic distances, with R^2^ = 0.81 and p-value = 0.004, presenting a significant Isolation by Distance (IBD) pattern.

Temporal genetic differentiation was also tested by *F*_ST_ values (Fig. 2) and by DAPC (Fig. 3a,b), for those locations where data from more than one fishery season were available (Figs. 2, 3). The data revealed no significant temporal genetic changes in samples from Olhão (Portugal) within the period of 14 years (approximately 9 generations considering *O. vulgaris* life spam). No significant temporal differences were found between 2018 and 2021 (2 generations) for the rest of the locations, except for the samples from Puerto de Vega collected between 2007 and 2018, with respect to the last sampling collected during 2020–2021 in the same locality (21PV) (Figs. 2, 3).

**Figure 4:**
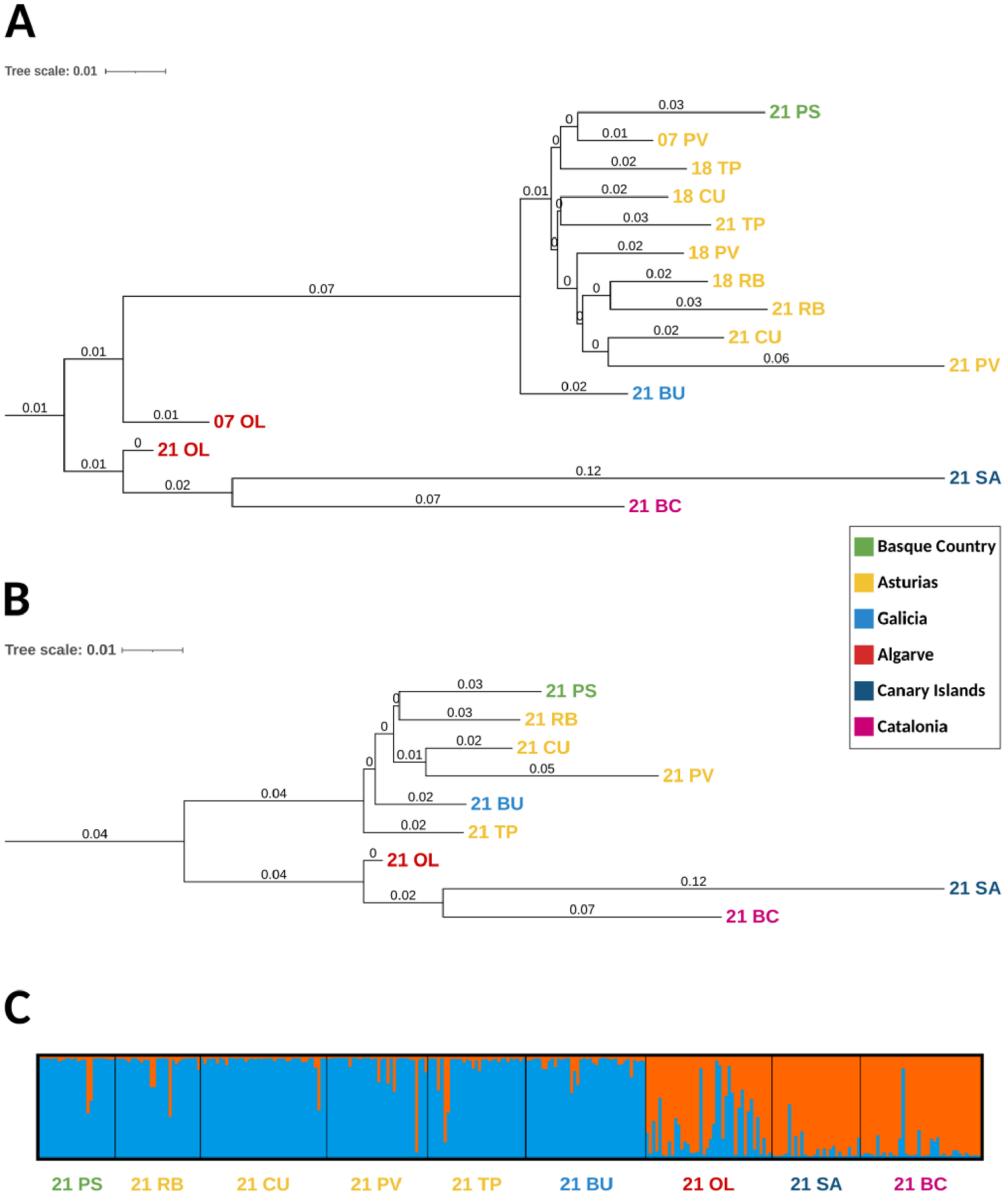
Neighbor-joining trees using *D*_*A*_ distance^57^ and bayesian analysis of *O. vulgaris* populations in this study. (**A**) Global analysis for the 9 localities showing all the fishery seasons, (**B**) global analysis for all localities showing only the 2020–2021 fishery season, (**C**) Structure bar-plot showing the assignment probabilities for each genotyped individual from 2020–2021 fishery season under admixture model. Each bar corresponds to one individual. Labels indicate the fishery season (first two digits) and the locality (Pasaia (PS), Ribadesella (RB), Cudillero (CU), Puerto de Vega (PV), Tapia de Casariego (TP), Bueu (BU), Olhão (OL), San Andrés (SA), Barcelona (BC)).

The Neighbour Joining tree obtained after Nei genetic distances D_A_ estimations^57^ clearly separated the Canary Islands and Barcelona from the rest of the samples, and separated Portugal from the rest of the other populations located further north (see the map in Figs. 1, 4a,b). The Bayesian analyses for structuring in *O. vulgaris* showed 2 main genetic clusters (Evanno’s k = 2, Lnʹ(K) = 733.240) (Fig. 4c) representing 2 ancestral lineages (i.e., Algarve, Canary Islands, and Mediterranean, and then the rest of Atlantic samples including the Bay of Biscay) (Fig. 4).

Effective population sizes were analyzed with NeEstimator 2.1^58^ using the temporal methods based on allelic variances^59^. The analyses showed estimates of N_E_-Puerto de Vega_(2007–2018)_ = ∞ (95% CIs for NE, Jackknife on Loci: 1042.5–∞); N_E_-Puerto de Vega_(2007–2021)_ = 182.3 (27.8–∞) and N_E_- Olhão_(2007–2021)_ = 1651.3 (292.7–∞) for approx. 7 to 9 generations (2007–2018–2021). The shorter period from 2018 to 2021 (approx. 2 generations) revealed the following estimates in Asturias N_E_-Ribadesella_(2018–2021)_ = ∞ (248.3–∞); N_E_-Cudillero_(2018–2021)_ = ∞ (261.5–∞); N_E_-Puerto de Vega_(2018–2021)_ = 46.6 (8.0–∞) and finally N_E_-Tapia de Casariego_(2018–2021)_ = 137.6 (54.6–∞).

## Discussion

This study aims to shed light on the genetic status of the populations of *Octopus vulgaris*, a species of great commercial interest, and on the efficiency of the current *O. vulgaris* management plan in Asturias, northern Spain.

### Genetic variation and management units (MU) for exploited *O. vulgaris* stocks in the eastern Atlantic area

Genetic diversity is the key element for maintaining a species’ potential to adapt to environmental changes, when there is undeniable evidence that the environment is changing mainly due to anthropogenic activity^60^. In this work, the levels of genetic variability found when using a set of 13 microsatellite markers on samples from 2007, 2018, and 2021 (mean K = 13.7, A_R_ = 7.0, H_E_ = 0.51, H_O_ = 0.49) did not show significant temporal changes. The reported values were just slightly lower than those previously reported by De Luca et al.^43^ (13 microsatellites, mean K = 15.8, A_R_ = 7.4, H_E_ = 0.65, H_O_ = 0.50) for Mediterranean *O. vulgaris* samples. Melis et al.^42^ (five microsatellites, Mean K = 27.6, A_R_ = 16.8, H_E_ = 0.90, H_O_ = 0.83) and Cabranes et al.^50^ (five microsatellites, Mean K = 18.3, H_E_ = 0.87, H_O_ = 0.75) reported higher genetic variation values for Mediterranean and Atlantic populations, respectively; but using a very low number of genetic markers. Significant loss of genetic variation will lead to loss of species’ evolutionary potential^61^. It is well established that overfishing is seen as the major threat to the loss of marine populations genetic diversity within populations and it has the potential (when fishing is highly selective) to permanently change the characteristics within a population, usually in directions of less economic value^62,63^. Negative consequences of losing genetic diversity will not only affect the fishing industry, but also disturb the respective predator–prey populations and eventually the entire marine ecosystem^64^.

The Hardy–Weinberg principle depends on a number of assumptions namely simple Mendelian inheritance in a diploid organism with discrete generations, random mating, an infinite population, and no mutation, migration, or selection^65^. None of the populations in our study showed significant deviations from the Hardy–Weinberg Equilibrium (HWE), and at the same time, no signals of recent bottlenecks were found in those localities under study. These results could suggest that those populations (all of them under fishery exploitation) are at rest and not perturbed in a significant way.

Our data includes a representation of the eastern Atlantic and Mediterranean area octopus populations (Basque Country, Asturias, Galicia, Portugal, Canary Islands, and Catalonia). The Bayesian analyses suggested two ancestral gene pools (1: Bay of Biscay and Galicia and 2: Algarve, Canary Islands, and Mediterranean areas). Contemporary genetic heterogeneity is evident at smaller geographic scales among Portugal, Canary Islands, and Mediterranean samples. Some samples (i.e. Puerto de Vega 2020–2021 in Asturias) showed peculiarities that revealed punctual genetic heterogeneity within Galicia and the Bay of Biscay samples analyses at very small distance scales (Figs. 1, 2). Note that, although the presence of null alleles (common when working with microsatellites) could lead to overestimation of both, *F*_ST_ and genetic distances^56^, our results remain the same when using the ENA correcting method. Previous studies on genetic structuring in *O. vulgaris* have been carried out based on mitochondrial and nuclear DNA datasets in the Mediterranean^42,43,49,66,67^ and Atlantic areas^31,49–51^. Using both types of markers, De Luca et al*.*^43^ found a pronounced differentiation of the Atlantic and Sicilian specimens supporting the isolation of the biota within the Strait of Messina and significant differentiation within the Mediterranean Sea as was previously suggested by Casu et al*.*^66^. Moreover, Melis et al*.*^42^ highlighted high variability and low but significant genetic differentiation among populations in a very small geographic scale in the Mediterranean, where samples clustered into four groups in the coasts of Sardinia. In the Atlantic area, significant subpopulation structure was also identified using only five microsatellites, consistent with an isolation-by-distance (IBD) model for Atlantic populations, but the genetic differences between pairs of samples separated by < 200 km were not significant^50^. Recently, Quinteiro et al*.*^51^ used mitochondrial DNA and suggested a significant differentiation in their study including insular and continental samples from the Galicia and Morocco coasts, with the exception of pairwise comparisons for samples from Madeira and the Canaries populations. These results pointed out the existence of genetically differentiated octopus populations in the Mediterranean and Atlantic areas and the necessity of local regulations for the appropriate management of octopus stocks. The octopus stocks of the Bay of Biscay had not been genetically monitored for more than fifteen years until the present study.

Our study area is also located in the Canaries-Iberian upwelling system. Even when upwelling was early viewed as dispersive environments for the larval stages, these systems are now considered more retentive areas than previously thought, where the larvae are capable to regulate their transport by exploiting the circulation patterns and later recruiting close to their natal habitats^68^. Genetic similarity within marine species is directly related to individual dispersal capability^31^. The movements of adult *O. vulgaris* have been found highly limited and within 1 km in most of the recaptures (84–86%) in a capture-recapture experiment conducted by Mereu et al.^69,70^ where high site fidelity and the necessity for creating small no-take areas were suggested. While it seems that planktonic *O. vulgaris* paralarvae have an oceanic strategy in upwelling systems rather than the coastal-shelf strategy of other neritic species (Loliginidae and Sepiidae families)^71^; pre-recruits usually are distributed in specific and differential grounds, as has been found in Portugal (8 pre-recruit grounds), where its western zone adjacent to Ria Formosa lagoon (southern coast) was identified as the main recruitment ground for *O. vulgaris* along the Portuguese coast^72^. Our Portuguese samples were coming from this area (Algarve) and as it is mentioned above, they showed high levels of genetic diversity. It seems also that environmental factors are fundamental to the behavior of the dispersal ability of paralarvae in *O. vulgaris*^73^ and wide inter-annual fluctuations in *O. vulgaris* abundances have been described in distinct geographic areas in connection with environmental drivers (reviewed by Roa-Ureta et al*.*^46^). In Galicia, it seems that a large fraction of the annual variability in catch is due the impact of upwelling on the survival of planktonic life stages^74^. Characterizing possible local paralarvae retentions in the Asturian coast as a consequence of hydrographic conditions as well as identifying recruitment grounds are still pending. Moreover, it had been recently argued that the Asturias *O. vulgaris* stock presents a rich dynamic that results from intrinsic properties of the stock, as well as from small perturbations from a combination of moderate fishing removals and possibly environmental forces that determine strong density-dependent and overcompensations that cause fluctuations in *O. vulgaris* stocks abundance^46^. These facts can explain punctual genetic heterogeneity within the Bay of Biscay area, where we have found a global pattern of genetic homogeneity (considering Basque Country, Asturias, and even Galicia) which is congruent with previous studies on the area (i.e.: Cabranes et al*.*^50^). Despite this, periodic genetic monitoring on these exploited and fluctuating stocks seems to be advisable. Deeper studies using genomic tools (i.e.: SNP studies covering wider areas within the octopus genome) will help in the future to re-assess structuring and management units in this small geographic area.

### The relevance of temporal genetic data on assessments for the MSC eco-labeled sustainable fisheries of the Asturias *O. vulgaris* fishing stock

In this work, we have found stable levels of genetic variation for the localities where temporal analyses were possible (Ribadesella, Cudillero, Puerto de Vega, Tapia de Casariego, all of them located in Asturias; and Olhão, which is located in Portugal). It is worth mentioning that temporal replicates were all in HWE. Temporal analyses are a powerful tool in population genetics and in its application to relevant problems, such as assessments of fishery stock status. Temporal studies allow to discerning between real genetic signals and noise artifacts, meaning that genetic patterns that are consistent through time are unlikely to be sampling artifacts^59^. Moreover, sampling adult population over generations and looking for statistically significant shifts in allelic frequencies that cannot be explained by evolutionary forces, such as mutation, selection, migration, or varying year-class strengths in populations, help to identify Sweepstakes Reproductive Success (SRS) patterns, common in marine species, where extremely large variance in individual reproductive success is due to sweepstakes-like chances of matching reproductive activity with oceanographic conditions^75^. In addition, temporal replicates allow estimating of effective population sizes, which is a relevant parameter that gives clues about the health state of an endangered/exploited population determining the rate of loss of genetic diversity, fixation of deleterious alleles, and the efficiency of natural selection at maintaining beneficial alleles^76^.

In the Portuguese samples (Olhão, Algarve, Portugal), the temporal replicates were genetically homogeneous and showed high diversities. Besides being a relevant recruitment zone, it seems that a relevant co-management plan has been reported in the area and has shown promising results for the sustainable use of fishery resources^77,78^. *O. vulgaris* stock assessment is difficult due to distinct life history features such as short life cycles, semelparous reproduction, high natural mortality rates, rapid growth, and complex population structures^79^. A keystone in any attempt at species conservation and/or management is the effective size of a population (N_E_). Fifty individuals (N_E_ = 50) have been considered necessary for a population’s immediate survival avoiding inbreeding depression^80^. In the Algarve, Portugal, high effective population sizes N_E_-OL_(2018–2021)_ = 1651.3 (292.7–∞) were found. In three out of the four Asturias locations temporally sampled in this work, our data revealed also high effective population sizes (N_E_-Ribadesella _(2018–2021)_ = ∞ (248.3–∞); N_E_-Cudillero _(2018–2021)_ = ∞ (261.5–∞); and N_E_-Tapia de Casariego _(2018–2021)_ = 137.6 (54.6–∞)). The decrease in the effective population size in the case of Puerto de Vega is the only noticeable case, as in the time range between 2007 and 2018 the value is infinite (N_E_-PV_(2007–2018)_ = ∞ (1042.5–∞)) while from 2018 to 2021, it drops sharply to 46.4 (N_E_-PV_(2018–2021)_ = 46.6 (8.0–∞). It has been said that octopus fisheries in Asturias exercise a low pressure on the stability and renewal capacity of the stocks^47^. During the first few years of the management plan (2000–2001 to 2007–2008), total annual landings averaged 180 tons, and total annual effort normally exceeded 3000 days of *O. vulgaris* fishing, but later (2008–2009 to 2018–2019), landings decreased, averaging 102 tons, and effort decreased as well to less than 2000 days^46^. Recently, Roa-Ureta et al*.*^46^ estimated abundances for octopus’ stocks within the Asturias management plan using depletion models and reported densities ranging from 1250 to nearly 5000 per km^2^ considering the area of the fishing grounds off in Asturias (228 to 397 km^2^). Moreover, they predicted recruitment from spawning abundance observations for female spawning stock and recruitment dynamics in *O. vulgaris* in this area. These temporal analyses indicate significant oscillations in fishing efforts and recruitments (see Fig. 5b in Roa-Ureta et al*.*^46^). Moreover, taking into account the entire available time range (2007–2021), the N_E_-PV_(2007–2021)_ = 182.3 (27.8–∞) is still high. The case of Puerto de Vega can be punctual, or an indicator of restricted gene flow^81^, or a signal about current environmental and/or fishery pressures on the specific zone perturbing the octopus populations. Discerning this will need more studies, including replication, to be evaluated.

## Conclusions

Findings from this study can give a better vision of the spatial and temporal distributions of genetic variation in common octopus in the Atlantic area, and of the efficiency of the current *O. vulgaris* management plan in Asturias, as well as of the healthiness levels of these fishery stocks. The Atlantic *O. vulgaris* populations show significant genetic structuring at a large geographical scale that fits with a classical isolation by distance model, where the probability of individuals mating with one another is restricted and local retention of paralarvae makes populations small in comparison to the total species distribution. That reinforces the necessity of local and regional plans to guarantee long-term sustainability. Results from our work can provide a baseline for further genomic studies on Asturian common octopus and therefore for sustainable exploitation. To the best of our current knowledge the Asturias *O. vulgaris* fishery plan seems to be currently adequate, since our data is not detecting recent harms to the fished stock and, accordingly, the validity of the MSC label seems to be rational. More data will be needed to assess if the Asturias management plan may require a more specific regional approach including smaller spatial scales.

## Materials and methods

### Ethics declaration

No use of live animals was required for this study. All samples used for the present study came from animals fished for commercial purposes or from the collections of other research centers. For more information, please see the Acknowledgements section.

### Samples, DNA extractions, and microsatellite amplifications

A total of 539 *O. vulgaris* individuals were collected in nine localities across the Bay of Biscay (Pasaia in the Basque Country, Spain; Ribadesella, Cudillero, Puerto de Vega, and Tapia de Casariego in Asturias, Spain), Portuguese waters (Bueu in Galicia, Spain; Olhão in Algarve, Portugal), Macaronesia (San Andrés in Canary Islands, Spain) and the Mediterranean sea (Barcelona in Catalonia, Spain) during the last fishery seasons (2020–2021) (Fig. 1).

Moreover, samples from the fishery campaign 2006–2007 from Portugal and Asturias (Puerto de Vega) and from the season 2017–2018 in Asturias (Tapia de Casariego, Puerto de Vega, Cudillero, Ribadesella) were also available for analyses. All the procedures were conducted using preserved or freshly small tissues from specimens collected by local fishermen and fixed in pure ethanol (100%). No use of live animals was required for this study.

DNA was extracted using EZNA® Mollusc Kit (Omega Bio-Tek Inc., Norcross, GA, USA). Approximately 25 mg from octopus tissues were cut and chopped into small pieces, put into 1.5 ml microcentrifuge tubes and processed following the manufacturer’s instructions. The resulting DNA was visualized on agarose gel 1% and stored in a − 20 °C freezer for further applications. Thirteen microsatellite loci were amplified reliably and arranged into two multiplex PCRs using Multiplex Manager 1.2 software^82^ according to dye colors and expected amplicon sizes. Microsatellite amplifications were carried out by combining 13 loci (previously tested in single PCRs) in two multiplex PCR reactions (Table 1), using QIAGEN Multiplex PCR Kit (QIAGEN Inc., Venlo, Netherlands) at the following conditions: 15 min at 95 °C, 40 cycles at 94 °C for 30 s, 55 °C for 1 min and 30 s, 72 °C for 1 min, and a final extension at 60 °C for 30 min. Each PCR reaction was conducted in a final volume of 13 μl. Forward primers were 5’ labeled using fluorescent dyes: 6-FAM™, NED™, VIC®, and PET® (Applied Biosystems, Foster City, CA, USA) (Table 1). PCR products were run on the Automated Capillary Electrophoresis Sequencer 3130XL Genetic Analyzer (Applied Biosystems) after a 1:10 dilution.

### Genetic variation

Microsatellite genotyping was conducted locus per locus by two different and independent readers using the automatic procedure implemented in Geneious Prime® 2020.2 and manually corrected. Possible genotyping errors and null allele frequency estimates were determined using Micro-Checker 2.2.3^83^ and FreeNA^56^, with the number of replicates fixed to 10,000. The data set corrected for null alleles was used as a final input file for further statistical analysis. Allele frequencies, the number of alleles per locus (k), the mean number of alleles (N_A_) per locus, the observed heterozygosity, within-population gene diversity and the overall gene diversity (H_O_ and Hs, and H_T_ respectively), were calculated using “adegenet”^84^, “pegas”^85^ and “hierfstat”^86^ packages implemented in R version 4.1.2 through RStudio 2021.09.2 + 382 “Ghost Orchid” Release. The “PopGenReport” package^87^ in R was used to calculate the allelic richness (A_R_) per locus and per population and also the number of private alleles (A_P_) within populations. Spatial and temporal comparisons for levels of genetic variation were conducted using a two-sided statistical analysis included in the FSTAT 2.94 software^88^ for several statistics (A_R_, H_O_, H_E_).

### Spatial and temporal genetic differentiation and clustering analyses

The *F* statistics following Weir and Cockerham^55^ and possible deviations from expected proportions in Hardy Weinberg’s equilibrium for each locus and population were assessed using FSTAT 2.94 software^88^. Significance levels of *F*_IS_ were estimated by permutating alleles between genotypes within samples 10,000 times, and adjusted following a Bonferroni correction^89^. Additionally, *F*_ST_ (ENA) values were estimated using FreeNA, which estimated the unbiased *F*_ST_ following the ENA method^56^. The bottleneck hypothesis was investigated using the program BOTTLENECK v 1.2.02 under the two-phased model of mutation (TPM)^90^, taking into account 90% single stepwise mutations with a variance of 12. The “Wilcoxon sign-rank test” was used to determine whether a population exhibits a significant number of loci with heterozygosity excess^91^. Pairwise *F*_ST_ values between samples and corresponded p-values were calculated using FSTAT 2.94 software where for significance levels of *F*_ST_, multi-locus genotypes were randomized between pairs of samples (10,000 permutations) and calculated after Bonferroni correction^88,89^. Comparisons between regions and between fishery seasons were conducted using a two-sided statistical analysis included in the FSTAT software for several statistics [A_R_, H_O_, H_E_, *F*_IS_, *F*_ST_, relatedness (R), and corrected relatedness].

A discriminant analysis of principal components (DAPC) using the R working package “adegenet”^84^, was conducted to cluster the samples in groups. Besides this, the population structure was also assessed with Bayesian clustering population structure analysis in STRUCTURE 2.3.4^92^. In order to fasten the procedure, structure analysis was performed through “ParallelStructure” package^93^ in R. Structure analysis was run among all nine populations, without taking temporal data into account. The settings used were an admixture model from K = 1 to K = 18 in 20 runs^94,95^. Assignment clusters were made with a length burn-in period of 20,000 and 200,000 Markov Chain Monte Carlo repetitions. The most likely value of K was chosen using the delta K statistic^94^, using the STRUCTURE HARVESTER software^96^. A Neighbour‐Joining (NJ) tree based upon pairwise Nei's genetic distance D_A_^57^ was constructed with the software POPTREEW^97^ using 10,000 bootstraps and visualized in The Interactive Tree of Life (https://itol.embl.de)^98^.

The Package “ade4”^99^ was used to perform a partial Mantel test to study if the observed patterns of genetic structure found here conformed to the isolation by distance model (IBD) explaining genetic isolation between populations^100^. The geographic distances between each pair of sampling localities (Kms) were calculated on the basis of a spherical earth (ignoring ellipsoidal effects), using the haversine formula^59^ and they were related with Edwards' distance^101^. The software BayeScan 2.1.^102^ was used to identify candidate loci deviating from neutral expectations from genetic data, using differences in allele frequencies between populations. Twenty pilot runs of 5000 iterations each, followed by an additional burn-in of 50,000 iterations, and then 5000 samplings with a thinning interval of 10 were conducted. Loci with α-value significantly > 0 and q-values < 0.05 were defined as “outliers”—i.e., loci putatively under directional selection. Loci with α-value significantly < 0 were considered putatively under balancing selection. The remaining loci were classified as neutral.

### Effective population sizes

Finally, temporal change in allelic frequencies was used for estimating the effective population sizes (N_E_) using the Temporal Method, Nei/Tajima, Plan II^103^ with the NeEstimator software^58^. The lifespan of *O. vulgaris* from several regions in the Atlantic frequently exceeds 1 year and may reach a maximum of nearly 2 years^104,105^. A generation interval of 1.5 years for *O. vulgaris* was used for N_E_ estimations, consequently, the 2007 fishing season was considered as the starting year (generation 0), while 2018 was considered generation 7 and 2021 generation 9.

## Data Availability

The datasets generated and/or analyzed during the current study are available in the Zenodo repository: 10.5281/zenodo.6635759. Microsatellite markers (GenBank accession numbers, [reference]): OCT08 (AF197132^50^); VULG15 (LC003035^51^); VULG14 (LC003034^51^); VULG07 (LC003028^51^); OVUL10 (JN579699^52^); VULG12 (LC003032,^51^); VULG13 (LC003033^51^); VULG06 (LC003027^51^); OVUL09 (JN579698^52^); VULG04 (LC003026^51^); OVUL08 (JN579697^52^); OV10 (AF197134^50^); VULG10 (LC003030^51^).
